# Fructose Containing Sugars at Normal Levels of Consumption Do Not Effect Adversely Components of the Metabolic Syndrome and Risk Factors for Cardiovascular Disease

**DOI:** 10.3390/nu8040179

**Published:** 2016-03-23

**Authors:** Theodore J. Angelopoulos, Joshua Lowndes, Stephanie Sinnett, James M. Rippe

**Affiliations:** 1 Obesity Research Center, School of Health Sciences, Emory & Henry College, 601 Radio Hill Rd, Marion, VA 24354, USA; 2 Rippe Lifestyle Institute of Florida, 215 Celebration Place, Celebration, FL 34747, USA; lowndesjoshua@hotmail.com (J.L.); comssinnett@rippelifestyle.com (S.S.); jrippe@rippelifestyle.com (J.M.R.); 3 Rippe Lifestyle Institute, 21 North Quinsigamond Avenue, Shrewsbury, MA 01545, USA; 4 Biomedical Sciences, University of Central Florida, 4000 Central Florida Boulevard, Orlando, FL 32816, USA

**Keywords:** fructose, high fructose corn syrup, sucrose, metabolic syndrome, cardiovascular disease

## Abstract

The objective of the current study was to explore our hypothesis that average consumption of fructose and fructose containing sugars would not increase risk factors for cardiovascular disease (CVD) and the metabolic syndrome (MetS). A randomized, double blind, parallel group study was conducted where 267 individuals with BMI between 23 and 35 kg/m^2^ consumed low fat sugar sweetened milk, daily for ten weeks as part of usual weight-maintenance diet. One group consumed 18% of calories from high fructose corn syrup (HFCS), another group consumed 18% of calories from sucrose, a third group consumed 9% of calories from fructose, and the fourth group consumed 9% of calories from glucose. There was a small change in waist circumference (80.9 ± 9.5 *vs.* 81.5 ± 9.5 cm) in the entire cohort, as well as in total cholesterol (4.6 ± 1.0 *vs.* 4.7 ± 1.0 mmol/L, *p* < 0.01), triglycerides (TGs) (11.5 ± 6.4 *vs.* 12.6 ± 8.9 mmol/L, *p* < 0.01), and systolic (109.2 ± 10.2 *vs.* 106.1 ± 10.4 mmHg, *p* < 0.01) and diastolic blood pressure (69.8 ± 8.7 *vs.* 68.1 ± 9.7 mmHg, *p* < 0.01). The effects of commonly consumed sugars on components of the MetS and CVD risk factors are minimal, mixed and not clinically significant.

## 1. Introduction

CVD is the single largest cause of mortality worldwide [1]. In the United States, despite some success in lowering the burden of CVD, it still represents the largest single source of death accounting for 37% of all mortality in 2013 [2].

The MetS represents a constellation of findings related to dyslipidemia, dysregulated glucose handling, hypertension and abdominal obesity [3,4,5,6,7,8]. While the prevalence of the MetS varies widely according to the various criteria used to define it, the prevalence has increased dramatically in the past 20 years. It has been estimated that up to 36% of the population in the United States meet the National Cholesterol Education Program (ATP-3) (NCEP) [8] criteria for MetS.

While multiple underlying risk factors for both CVD and MetS have been identified, nutritional patterns certainly play a significant role. In the past, emphasis on the relationship between nutritional practices and these metabolic diseases has focused largely on the intake of certain types of fat (e.g., saturated fats and trans fat) [9], as well as overall caloric consumption. Recently, dietary sugars have also come under scrutiny because of epidemiologic studies suggesting a possible link between intake of fructose containing sugars such as high fructose corn syrup (HFCS) and sucrose and risk factors for CVD [10,11,12,13] and components of the MetS [11,14,15]. These studies have focused largely on the relationship between fructose containing sugars and established risk factors for CVD and components of the MetS such as dyslipidemia, elevated blood pressure, obesity, abdominal fat and glucose [16,17,18,19,20,21].

Consumption of added sugars has increased somewhat in the United States since 1970. Between 1970 and 1984 energy intake and consumption of added sugars increased from approximately 325 kcals to approximately 350 kcal/day per person [22]. Between 2005 and 2010 the number of calories from added sugars declined to 326 kcals per person per day [23]. One-third of this decrease resulted from decreased sugar sweetened beverage (SSB) consumption.

Some but not all studies have linked excess sugar consumption to increases in risk factors for CVD and components of the MetS. For example, some studies have linked consumption of high levels of fructose containing sugars (levels greater than the 90th percentile population consumption) to dyslipidemias including increased LDL cholesterol (LDL), decreased LDL particle size, and increase in TGs [24,25,26,27]. Some studies have linked excessive sugar consumption to increased blood pressure [28,29,30] and inflammation [31,32,33], although other trials in this area have been inconclusive [34]. Several studies comparing fructose to glucose consumption have reported increases in abdominal fat [35,36]. The American Heart Association has recently summarized links between sugar consumption and risk of CVD and recommended that the average adult male not consume more than 150 kcal/day from all added sugars and that the average adult female not consume more than 100 kcal/day in added sugars [10]. These recommendations are currently exceeded by over 80% of the adult population in the United States.

Whether there is a unique link between fructose and metabolic diseases remains highly controversial. The current study was undertaken to see if consumption of fructose containing sugars at the average consumption levels in the United States (50th percentile population consumption levels for fructose based on data from Marriott *et al.*) [37] resulted in increased risk factors for CVD and levels of MetS components. Three fructose containing sugars, namely fructose itself, sucrose, and high fructose corn syrup (HFCS) consumed at average consumption levels for fructose were delivered in 1% low-fat milk and compared to a control condition of glucose sweetened low-fat milk delivered at comparable caloric consumption levels. Based on previous work in our research laboratory and recently systematic reviews and meta-analyses by Chiavaroli *et al.* [38] and Wang *et al.* [39] we hypothesized that average consumption levels of these sugars would not adversely impact risk factors for CVD and MetS components and that there would be no significant differences between fructose containing sugars and glucose on these risk factors. To explore this hypothesis, we asked subjects to consume three different fructose containing sugars (HFCS, sucrose and fructose) at the 50th percentile population consumption level of fructose and compared their responses after 10 weeks of consumption to glucose consumption matched for calories with the fructose only arm.

## 2. Experimental Section

### 2.1. Study Design and Participants

This was a randomized, prospective, parallel group, double blind study to examine the effects of consuming 18% of calories from sucrose sweetened low-fat milk *versus* 18% of calories from HFCS sweetened low-fat milk *versus* 9% of calories from fructose sweetened low fat milk and 9% of calories from glucose sweetened low-fat milk. The higher percentage of calories from HFCS and sucrose was chosen to equilibrate the levels of fructose consumption at 9% of calories since these two sugars are combinations of fructose and glucose. HFCS-55, which was utilized, contained 55% fructose, 42% glucose and 3% glucose polymers. Sucrose contains 50% fructose and 50% glucose. Previous studies from our research laboratory demonstrated that these two sugars have equivalent effects on a variety of nutritional and metabolic parameters [40,41,42].

The level of 9% of calories from glucose was chosen to equilibrate calories with the fructose condition. The low-fat sweetened milk was consumed as a component of a subject’s usual diet. Low fat milk was chosen because of its relatively neutral pH. Previous studies conducted in our research laboratory and others have been criticized for utilizing carbonated soft drinks where a significant amount of inversion of sucrose can occur. Indeed, the process of sucrose inverting into glucose and fructose is well described and is affected by both temperature and pH [43]. One recent study exploring fructose levels showed that most of the sucrose had inverted prior to the start of the study and all of it had inverted by the end of the study. To minimize the prospect of inversion and ensure that we were actually studying HFCS *vs.* sucrose *vs.* fructose *vs.* glucose, our sugar sweetened milk samples were tested by a blinded, outside analytical laboratory (Archer Daniels Midland, Decatur, IL, USA). These studies showed that no significant inversion of the sucrose had occurred over the course of the study. In addition, the sweetened low fat milk containers were scrupulously kept in a refrigerated warehouse and refrigerated facilities at our research laboratory to prevent any issues related to temperature which might catalyze inversion.

A further consideration involved the fact that we were asking individuals to consume these sugar sweetened beverages for a period of ten weeks. We felt it was more ethically correct to have individuals consume these added levels of sugars in milk, which gave some nutritional benefits.

**Figure 1:**
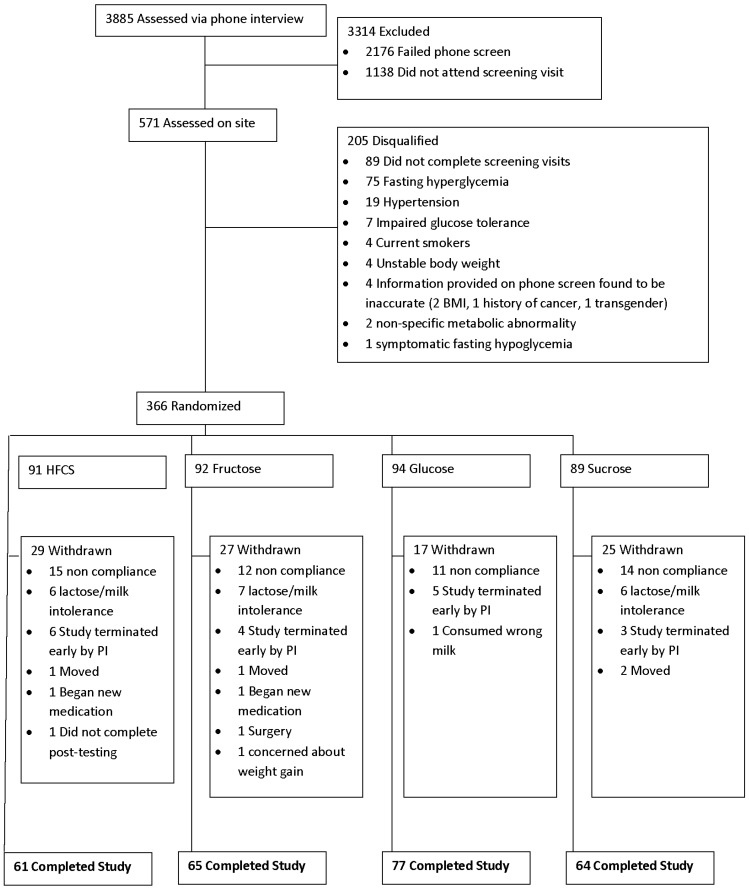
Participant flow over the course of the ten-week trial: 366 participants were randomly assigned to one of four groups, each required to consumed a low-fat, sugar-sweetened milk in an amount that the added sugar contributed 9% (fructose and glucose) or 18% (sucrose and high fructose corn syrup (HFCS)) of the daily energy intake required for weight maintenance.

Three hundred and sixty-six (366) otherwise healthy, normal weight, overweight, and obese subjects (both men and women; 131 men and 235 women) between the ages of 20 and 60 years old with Body Mass Index (BMI) between 23 and 35 kg/m^2^ were recruited for the study. The recruitment and randomization schematic is depicted in Figure 1. Both staff members and subjects were blinded as to whether or not participants in the trial were consuming sucrose, HFCS, fructose or glucose sweetened milk. Staff members, however, were aware of whether the subjects were consuming 18% of calories from added sugar (either HFCS or sucrose in a blinded fashion) or 9% of calories from added sugar (either fructose or glucose) since this information was needed in order to prescribe the remainder of the diet. While they were blinded to the actual sweetener in the milk, subjects were nonetheless counseled in private counseling rooms in individual sessions to avoid the possibility of subjects discussing their nutritional intervention (e.g., sweetness of the milk, number of cartons prescribed, *etc.*) with individuals in other groups. The duration of the study was ten weeks for each participant and $300 compensation was provided for those who completed the full ten weeks. The study was approved by the Western Institutional Review Board (Puyallup, WA, USA) and all subjects signed informed consent forms. The study was conducted at our two research facilities in Florida—one in Orlando, Florida and the other in Celebration, Florida. The study was performed in accordance with ethical standards laid down in the 1965 Declaration of Helsinki and its later amendments. This trial has been registered with the NIH Clinical Trial Registry: NCT01797042.

Individuals were excluded if they were currently enrolled in a commercial weight loss program or taking weight loss medication or supplements. Individuals could not have experienced a weight change greater than five pounds in the prior 30 days and could not be using tobacco products or have utilized such products within the past year. Individuals diagnosed with type 1 or type 2 diabetes, uncontrolled hypertension (≥140/90 mmHg) were also excluded. Individuals who had thyroid disease and either stopped taking medication or had a dosage change within the prior six months were excluded. Individuals with a history of major surgery within three months prior to enrollment or a history of heart problems (e.g., myocardial infarction, bypass surgery, *etc.*) within three months of enrollment were also excluded. Individuals with orthopedic limitations that would interfere with moderate, regular physical activity were excluded. Individuals with a history of a surgical procedure for weight loss or gastrointestinal disorders or diagnosed eating disorders, history or presence of cancer or congestive heart failure were also excluded. Any individual consuming ≥14 alcoholic drinks per week was excluded as were women who were pregnant, lactating or trying to become pregnant. Individuals with a known allergy to sucrose, HFCS, fructose or glucose or participation in another clinical trial within 30 days prior to enrollment as well as lactose intolerance or any other significant food allergy were also excluded.

A standardized screening form listing the inclusion and exclusion criteria for the study was utilized to initially screen interested individuals over the phone to determine eligibility based on self-reported data. A phone script containing this information and the requirements for participation in the study was employed to ensure that screening occurred in a consistent manner. Self-reported data, including height and weight, were verified during the initial clinical visit. Fasting blood samples were obtained for lipids, insulin, glucose and C-reactive protein (CRP). Following the ten-week intervention another fasting blood sample was also obtained. All cholesterol samples were sent to a certified research based laboratory (Clinical Pathology Laboratory, Orlando, FL, USA) with error rates less than one percent.

Measurements for components of the Metabolic Syndrome were taken in the morning in a fasted state using standard procedures both before and after the ten week intervention. Resting Blood Pressure was measured prior to a blood sample being obtained using a standard Serum Separation Tube (Becton, Dickinson and Company, Franklin Lakes, NJ, USA). In addition to components of the Metabolic Syndrome (glucose, triglycerides and HDL), the serum was also used for the measurement of total cholesterol, LDL and C-reactive protein (CRP). 

### 2.2. Dietary Plans

During the second screening visit prior to the intervention, research dietitians assessed baseline participant dietary intake analyzing a completed three-day food record using the Nutrient Data System Research (NDS-R) software (University of Minnesota, Minneapolis, MN, USA). Following the ten-week intervention a repeat three-day food record was obtained and analyzed.

Following the completion of the two qualifying visits, individuals were randomly divided into four groups.

Group 1: Sucrose sweetened milk so that the sucrose provided 18% of the calories required for weight-maintenance (50th percentile of population consumption of fructose)

Group 2: Fructose sweetened milk so that fructose provided 9% of calories required for weight-maintenance (50th percentile of population consumption of fructose)

Group 3: HFCS sweetened milk so that the HFCS provided 18% of calories required for weight-maintenance (50th percentile of population consumption of fructose)

Group 4: Glucose sweetened milk so that glucose provided 9% of the calories required for weight-maintenance (control condition)

All sweeteners were supplied in 1% low-fat milk (Tetra-Pak, Denton, TX, USA). The caloric targets for weight-maintenance were estimated using the Mifflin-St. Jeor equation for REE (Resting Energy Expenditure) with activity factor.

Research dietitians prescribed the sweetened products on a weekly basis in amounts appropriate for each participant’s calorie intake level. The milk was provided in 8-ounce containers with either 15 g of added sugar (fructose or glucose condition) or 30 g of sugar (HFCS or sucrose conditions). Subjects consumed between 2 and 6.5 cartons/day depending on their calorie intake level. Subjects were allowed to drink their allotted quantity of milk at whatever times of day fit most conveniently into their daily life. Participants were also given specific limitations on the ways in which the milk could not be used. They were instructed not to cook with it or heat it (e.g., adding to dry oatmeal prior to cooking), to not use it to make yogurt, and to not add any ingredients that might influence how the sugar is metabolized (e.g., cocoa).

Counseling sessions were given to all subjects by registered dietitians at the time of initiation and weekly thereafter. Diet checklists were used by subjects to monitor appropriate consumption of food and beverage each day. The weekly dietary checklists were reviewed by dietitians at each counseling session. The subjects were encouraged to discuss challenges and compliance was emphasized.

### 2.3. Statistical Analysis

Power calculations were performed to account for a dropout rate of 33%. The requirement of sample size was determined by performing power calculations on TGs—the most variable of the measures included in the study. The observed dropout rate of 27% is consistent with what we have experienced in previous, similar studies.

Data were checked for normalcy and analyzed using a 2 (time) × 4 (group) × 2 (gender) analysis of variance with repeated measures. Only data on those subjects who completed the intervention were included in the analyses. Significant time by group assignment interactions were probed by assessing the within subject change for each of the four groups independently. In addition, changes over the course of ten weeks (Week 10 minus baseline) were calculated and between group differences assessed by one way ANOVA. Values presented are means ± standard deviations (S.D.). For analyses the alpha was set at 0.05. All data were analyzed using SPSS Advanced Statistics V18 (Chicago, IL, USA).

## 3. Results

### 3.1. Baseline Subject Characteristics

Of the 366 subjects who started the study, a total of 267 completed the 10-week trial (27% drop out) (see Figure 1). Individuals dropped out for a variety of reasons including moving away from the area, dissatisfaction with the protocol, were lost to follow up or were withdrawn from the protocol by investigators for failure to comply with aspects of the protocol including consuming the required number of milk servings. Those individuals who dropped out or were withdrawn were not different in any clinical parameter from those who completed the trial. Only data from the 267 individuals who finished the trial are included in this report. Of the 267 participants who completed the study, 96 were males and 171 were females. Subjects were on average 37.7 + 12.1 years old and had a BMI slightly above the healthy weight range (average BMI = 26.3 + 3.3 kg/m^2^). There were no significant clinical differences amongst individuals at baseline in any of the four groups. Data on the 267 individuals who finished the trial are presented in Table 1.

### 3.2. Dietary Intake

Compliance with consumption of the sweetened milk in all groups was very high with >95% of prescribed servings consumed over the ten week intervention. Compliance was measured using daily food checklists that were reviewed on a weekly basis with each subject by a research nutritionist. Energy intake increased in the entire cohort (*p* < 0.001), although consistently among the groups (interaction *p* > 0.05) Fat intake as a percentage of total calories decreased over the ten-week intervention in the entire cohort and comparably among all four groups (time *p* < 0.001, interaction *p* > 0.05). Percent calories from carbohydrate intake increased overall in the entire cohort (*p* < 0.001). Increases in the HFCS were greater than those in the fructose and glucose groups, and increases in the Sucrose group were greater than those in the fructose group alone (*p* < 0.05 for all comparisons). These differences between the HFCS and sucrose groups compared to the glucose and fructose groups were not surprising given that these two groups were consuming twice as many kcal/day from added sugar. Protein intake did not change over the course of the ten-week intervention and was not different among the four groups (Table 1).

Total sugars increased from baseline to week ten in all groups (103.3 ± 52.7 g *vs.* 183.2 ± 59.2 g *p* < 0.001) and rose more in the HFCS (90.7 ± 56.0 g *vs.* 203.0 ± 56.9 g) and sucrose group (102.6 ± 49.2 g *vs.* 203.4 ± 53.7 g) than compared to the glucose (104.2 ± 43.3 g *vs.* 160.7 ± 51.2 g) and fructose (113.8 ± 50.9 g *vs.* 171.6 ± 63.6 g) groups (*p* < 0.001).

### 3.3. Components of the Metabolic Syndrome

There were increases in waist circumference (80.9 ± 9.5 cm *vs.* 81.5 ± 9.5 cm, *p* < 0.001) and TGs (11.5 ± 6.4 *vs.* 12.6 ± 8.9 mmol/L, *p* < 0.01) in the entire pooled cohort of 267 participants. The increase in TGs was gender dependent with an increase only being observed in men (12.7 ± 7.6 *vs.* 15.0 ± 12.5 mmol/L, *p* < 0.05), but not in females (10.8 ± 5.4 *vs.* 11.3 ± 5.7 mmol/L, *p* > 0.05). The increase in TG was also greater in the HFCS group than in the glucose group although the large Systematic error of measurement (SEM) for the HFCS group makes this result questionable. In contrast both systolic blood pressure (SBP) and diastolic blood pressure (DBP) were lower at week ten than at baseline in this pooled cohort (*p* < 0.001), while there were no changes in High Density Lipoprotein (HDL) or fasting glucose. None of the small changes observed were considered clinically significant. Other than TGs, no other components of the MetS were influenced by the type of sugars consumed or by gender (Table 2).

### 3.4. Cardiovascular Disease Risk Factors

There were small increases in the entire cohort in BMI (26.2 ± 3.2 *vs.* 26.5 ± 3.4, *p* < 0.001) and for total cholesterol (4.6 ± 1.0 *vs.* 4.7 ± 1.0 mmol/L, *p* < 0.01). However, the type of sugars consumed did not influence these changes (interaction *p* > 0.05). Furthermore, there were no changes in LDL or CRP (Table 3). Gender did not affect any CVD risk factor (*p* > 0.05).

## 4. Discussion

The results of this study confirmed our hypothesis that while some changes occurred as a result of consuming sugar-sweetened milk, consumption of fructose and other fructose containing sugars produced largely similar effects on MetS components or risk factors for CVD compared to consuming glucose when the sugars were consumed over a ten week time period at average consumption levels (50th percentile population consumption level for fructose) Furthermore, when consumed as a part of a normal diet at typical levels, the effects of these sugars on risk factors for CVD and MetS components are small and mixed and do not carry clinical significance.

The effects of sugars on lipids have been the subject of multiple research trials [24,25,26,27,44,45,46,47,48,49,50]. These studies have typically employed levels of sugars above the 90th percentile of population consumption levels. Numerous studies have demonstrated that when carbohydrates replace fat, plasma TGs levels may increase, along with a decrease in HDL cholesterol. The type of carbohydrate appears, however, to play a significant role in generation of increased TGs. In diets that are high in sugar (>20% of energy), elevations in TGs may occur [47,49]. These increases appear to be more marked in men than women and also in individuals with the metabolic syndrome or those living a sedentary lifestyle.

Particular concerns have been raised that the fructose moiety in sucrose or HFCS may be particularly prone to contribute to elevated TGs levels because of differences in metabolism between fructose and glucose in the liver. It has been argued that these differences may result in hepatic TGs synthesis as well as *de novo* lipogenesis in the liver and reduced peripheral TGs clearance [47,48]. In the current study there were slight increases in triglycerides, (pre = 101.5 ± 56.4 mg/dL; post = 111.7 ± 79.1 mg/dL; *p* = 0.02). Further, changes in triglycerides following the 10 weeks intervention were greater in the HFCS compared to glucose group. This finding remains unexplained. It may be a result of the increased consumption of sugars in the HFCS group, although the sucrose group consumed a comparable amount and did not have increases in TGs. Nonetheless, simple sugars have been found to increase TGs and this may play a role in the difference between the pre and post HFCS group and the glucose group.

It should be pointed out, however, that all of these TGs levels were well within the normal population range and that none of these values was clinically significant. Furthermore, the issue of whether or not increases in TGs related to fructose containing sugars *per se* or increases in total energy must be taken into consideration. Chiavaroli *et al.* showed that when fructose was substituted isocalorically for other carbohydrates it did not have an impact on fasting TGs [38]. Wang *et al.* reported that when fructose was substituted isocalorically for other carbohydrates it had no differential effect or adverse effect on post-prandial TGs [39]. In the current study, energy intake increased over 200 kcal/day during pre-to-post measurements and weight increased by an average of approximately two pounds comparing pre-to-post measurements. Thus, the slight increases in TGs are more likely attributable to increased energy consumption or weight gain rather than any effect of fructose containing sugars *per se*. Furthermore, there were no differences in these parameters when comparing fructose containing sugars to glucose (interaction > 0.05). Furthermore, increases in TGs when comparing HFSC and sucrose to either glucose or fructose may have been the result of increased sugars consumption from the former two groups given that these groups had to consume twice as much sugar as the latter two groups in order to equilibrate the fructose level at 9%. The average BMI of our population was 26.27 ± 3.29, suggesting that these individuals on average were overweight and might be more prone to increased TGs in response to levels of added sugars employed in this research protocol. Thus, the findings from our study support the systematic reviews of Chiavaroli *et al.* [38], Wang *et al.* [39] and Sievenpiper *et al.* [51].

It should be pointed out, however, that both the pre and post levels were well within normal population standards. While there was a small increase in LDL when comparing the fructose containing sugars to glucose control condition nor were there any significant decreases in HDL when comparing the fructose containing sugars to glucose.

Furthermore, both the LDL and HDL levels were well within population norms. Thus, attributing any significant change in LDL or HDL to fructose containing sugars is not supported by our data.

Differences between our data and those of previous investigators may be due to differences in dosage level of fructose containing sugars, different types of sugar studied (*i.e.*, HFCS and sucrose *versus* fructose in previous studies) [33,36] or the population studied.

In particular, previous studies which showed increases in LDL often utilized dosages of fructose containing sugars at the 90th percentile population level or above or used pure fructose which is not consumed at any appreciable degree within the normal human diet. These issues have been the subject of several recent reviews [52,53]. Furthermore, studies utilizing pure fructose often employ very high doses of 25% kcal/day or higher which is greater than the 95% percentile population consumption of all fructose containing sugars in the normal human diet [35,36].

The slight increases in waist circumference and TGs would appear to be a result of the average two-pound weight gain that occurred in this cohort. While we attempted to create an environment where the added sugars would not create weight gain, we did not tightly control the free living environment, thus, a slight weight gain occurred. This indicates that people may have had difficulty incorporating the added sugars into their regular diet without inadvertently increasing caloric intake. This is an issue that was also reported by Stanhope *et al.* [35]. When these investigators conducted a trial similar in duration (10 weeks) where either added fructose or added glucose were given at 25% of calories, they too reported an approximately two-pound weight gain [35].

As already indicated, the increased average caloric consumption and weight gain in our study may have also contributed to increases in TGs, particularly, in the HFCS and sucrose group where twice as much added sugars were consumed as in the fructose or glucose groups.

In a previous study where we more tightly controlled the diets to be eucaloric, we noted that there was no increase in abdominal fat as measured by DEXA, nor was there any increase in TGs [40]. Moreover, the current findings are consistent with a series of systematic reviews and meta-analyses conducted by Sievenpiper and colleagues [54] which showed that there were no increases in weight when fructose containing sugars were substituted in an isocaloric fashion for other carbohydrates. Livesey and Taylor also reported similar findings in their meta-analysis [55]. Moreover, Cozma *et al.* conducted a systematic review and meta-analysis of fructose studies and found no increases in fasting glucose or insulin in trials where fructose was exchanged isocalorically for other carbohydrates [56]. Ha *et al.* reported no changes in blood pressure in studies where fructose was exchanged isocalorically for other carbohydrates [57]. Chiavaroli *et al.* showed that there was no increase in fasting TGs based on studies where fructose was exchanged isocalorically for other carbohydrates [38] and Wang *et al.* showed similar findings for post-prandial TGs [39]. Increases in weight [54] and TGs [38], however, occurred when fructose was added in hypercaloric trials. Thus, it would appear likely that the minor changes in TGs and waist circumference that we report were due to the slight increase in body weight.

Some [28,29], but not all [58] studies have suggested that high levels of intake of added sugars make contribute to elevations in blood pressure. Johnson *et al.* have proposed a mechanism through which fructose metabolism may result in increased levels of uric acid, which, in turn, may result in endothelial dysfunction and contribute to high blood pressure and MetS [12]. A recent systemic review and meta-analyses by Ha and colleagues involving 376 studies showed slight increases in both diastolic and mean arterial blood pressure when fructose was substituted hypercalorically for other carbohydrates [57] however, no increase occurred when fructose was substituted isocalorically. It should be noted that some clinical and experimental studies have shown that excessive intake in fructose increases blood pressure in both animals and humans. Jadall *et al.* Araujo *et al.* and De Angelis *et al.* all showed increases in blood pressure in response to fructose in animals; whereas, Stanhope *et al.* showed this finding in humans [59,60,61,62]. It should be noted that the handling of fructose in rodents may be quite different than in humans [61] (Sievenpiper *et al*. argued that this may be a story of mice but not men) [63]. Furthermore, the study by Stanhope *et al.* utilized fructose levels significantly higher than in the current study, which may account for the differences reported by these investigators.

In the current study, there were no differences in either systolic or diastolic blood pressure when comparing the three fructose containing conditions with the glucose condition. The slight decreases in both systolic and diastolic blood pressure may be associated with the fact that all of the sugars were delivered in 1% low-fat milk. This represents a potential confounder since the calcium, proteins or other components of milk may be associated with decreased blood pressure. Nonetheless, the current study does not support the contention that fructose containing sugars when consumed at normal population consuming levels increases blood pressure.

Several studies have suggested that high consumption of sugar sweetened beverages and foods may be associated with increased inflammation and oxidative stress [31,32,33]. Other studies, however, have not supported these findings [34]. In the current study there were no increases in hs-CRP when comparing pre-to-post conditions and when comparing fructose containing sugars to glucose.

It should also be noted that a recent study of dairy consumption in men and women with the metabolic syndrome, who consumed low fat dairy products for six weeks showed small and differential improvements in the metabolic syndrome markers for men and women [64]. Men had lower glucose (95.4 + 9.1 *vs.* 98.9 + 10.6 mg/dL, *p* = 0.04) while women had lower body weight, waist circumference and body mass index (*p* < 0.01) when compared to control. Thus, it is possible that the lack of extensive increases in components of MetS in the current study may be attributable to the consumption of low fat dairy. It should also be noted that 64% of the participants in this trial were women, which may further limit the implications of our findings.

The strengths of the current study include its relatively large sample size and the fact that it was a blinded, randomized, prospective study exploring normal population consumed levels of fructose from three different fructose containing sugars including the two most commonly consumed fructose containing sugars (sucrose and HFCS) compared to a glucose control. Weaknesses include that subjects were followed for only ten weeks and that subjects over the age of 60, children and adolescents were excluded and very few individuals below the age of 25 were studied. Adolescents represent a high fructose consuming group in the United States while young adults (aged 19–22 years old) represent the highest fructose consuming group [37].

## 5. Conclusions

The current study showed that there were minimal and mixed impacts on MetS components and CVD risk factors of fructose containing sugars in individuals consuming the 50th percentile of population consumption levels of fructose compared to glucose. Importantly, none of these changes were clinically significant. It should be emphasized that these results encompass a ten-week intervention period. Further longer term, carefully controlled studies perhaps utilizing higher levels of fructose containing sugars within the range of normal human consumption are needed. It is particularly important that these studies dealing with public health issues such as the MetS utilize the sugars which are commonly consumed in the human diet—namely, sucrose and HFCS—a factor that was emphasized in the recently released report from the National Institutes of Health (NIH) Workshop on Clinical Research Strategies for Fructose [65].

## Figures and Tables

**Table 1:** Changes in weight and dietary intake during ten weeks of daily consumption on each of 4 different types of sugar.

		HFCS	Fructose	Glucose	Sucrose	All
Age (Years)	Baseline	36.32 ± 10.72	38.65 ± 12.19	36.10 ± 12.06	39.83 ± 13.19	37.66 ± 12.11
Weight (Lbs)	Baseline	162.09 ± 26.12	166.61 ± 30.90	159.13 ± 24.88	161.66 ± 27.26	162.28 ± 27.28
	Week 10	164.05 ± 27.28	168.15 ± 32.52	160.44 ± 24.81	164.66 ± 27.99	164.15 ± 28.14 **
Energy Intake (Kcal)	Baseline	1929.4 ±733.7	2065.8 ± 722.9	1978.6 ± 644.3	2069.5 ± 711.6	2011.8 ± 708.2
	Week 10	2390.1 ± 700.7	2195.4 ± 581.7	2182.2 ± 636.6	2277.5 ± 668.8	2254.6 ± 647.6 **
Calories from Fat (%)	Baseline	32.6 ± 7.5	33.4 ± 12.4	33.7 ± 7.4	33.7 ± 6.5	33.7 ± 8.7
	Week 10	26.0 ± 5.5	28.1 ± 5.8	28.6 ± 6.0	25.2 ± 5.3	27.1 ± 5.8 **
Calories from Carbohydrate (%)	Baseline	47.8 ± 9.7	51.0 ± 13.5	47.8 ± 10.0	48.2 ± 7.9	48.7 ± 10.5
	Week 10	55.3 ± 6.6	52.3 ± 7.2 ^†,¥^	51.7 ± 7.9 ^†^	57.1 ± 5.7	54.0 ± 7.3 **
Calories from Protein (%)	Baseline	18.6 ± 6.8	18.3 ± 5.5	17.4 ± 5.1	17.0 ± 4.8	17.8 ± 5.5
	Week 10	18.3 ± 4.0	18.9 ± 3.4	19.3 ± 4.5	16.9 ± 2.7	18.4 ± 3.8

Note: HFCS—High fructose corn syrup, %—energy intake from the macronutrient as a percentage of total energy intake. All Groups consumed sugar sweetened, low-fat milk in amounts that the added sugar contributed 9% (fructose and glucose groups) or 18% (sucrose and HFCS groups) of the calories required for the participant to maintain weight. ** Significant difference pre *vs.* post in the entire study population *p* < 0.001; ^†^ Change significantly different than group HFCS *p* < 0.01; ^¥^ Change from baseline significantly different than group Sucrose *p* < 0.01.

**Table 2:** The effect of ten weeks of daily consumption on each of 4 different types of sugar on diagnostic criteria for the Metabolic Syndrome.

		HFCS	Fructose	Glucose	Sucrose	All
Waist circumference (cm)	Baseline	80.51 ± 9.29	82.06 ± 10.43	79.72 ± 8.47	81.45 ± 9.73	80.88 ± 9.45
	Week 10	81.31 ± 8.89	82.61 ± 10.97	79.84 ± 8.47	82.38 ± 9.43	81.45 ± 9.46 **
SBP (mmHg)	Baseline	108.61 ± 9.70	107.71 ± 10.51	109.17 ± 10.09	111.14 ± 10.28	109.16 ± 10.17
	Week 10	107.57 ± 11.29	105.49 ± 9.97	104.60 ± 9.78	107.25 ± 10.55	106.13 ± 10.39 **
DBP (mmHg)	Baseline	69.48 ± 9.71	69.68 ± 8.83	68.96 ± 8.33	71.14 ± 8.02	69.78 ± 8.71
	Week 10	68.90 ± 10.78	67.82 ± 8.91	66.12 ± 9.35	69.81 ± 9.48	68.05 ± 9.68 *
Triglycerides (mg/dL)	Baseline	98.20 ± 52.46	104.29 ± 68.24	100.28 ± 56.19	103.54 ± 47.56	101.56 ± 56.47
	Week 10	129.03 ± 120.49 ^†^	106.86 ± 66.13	99.86 ± 57.21	114.21 ± 60.43	111.70 ± 79.14 *
HDL (mg/dL)	Baseline	53.52 ± 13.07	51.45 ± 12.97	52.92 ± 13.06	52.11 ± 12.11	52.51 ± 12.77
	Week 10	53.08 ± 12.91	51.42 ± 12.75	52.33 ± 12.91	52.67 ± 12.40	52.36 ± 12.93
Glucose (mg/dL)	Baseline	89.43 ± 6.54	90.48 ± 7.07	90.66 ± 6.18	89.27 ± 6.27	90.00 ± 6.50
	Week 10	88.41 ± 8.24	91.65 ± 9.25	91.03 ± 6.69	91.38 ± 7.09	90.66 ± 7.89

Note: HFCS—High fructose corn syrup, SBP—Systolic Blood Pressure, DBP—Diastolic Blood Pressure, HDL—High Density Lipoprotein. All Groups consumed sugar sweetened, low-fat milk in amounts that the added sugar contributed 9% (fructose and glucose groups) or 18% (sucrose and HFCS groups) of the calories required for the participant to maintain weight. * Significant difference pre *vs.* post in the entire study population *p* < 0.01; ** *p* < 0.001; ^†^ Change from baseline significantly different than group Glucose *p* < 0.05.

**Table 3:** The effect of ten weeks of daily consumption on each of 4 different types of sugar on non-ATPIII Cardiovascular Risk Factor.

		HFCS	Fructose	Glucose	Sucrose	All
BMI (kg/M^2^)	Baseline	26.26 ± 3.16	26.52 ± 3.33	25.66 ± 3.14	26.75 ± 3.51	26.27 ± 3.29
	Week 10	26.58 ± 3.32	26.75 ±3.52	25.88 ± 3.17	27.24 ± 3.62	26.58 ± 3.42 **
Total Cholesterol (mg/dL)	Baseline	175.33 ± 41.67	181.26 ± 40.84	176.28 ± 39.35	176.75 ± 36.43	177.39 ± 39.44
	Week 10	179.98 ± 41.96	180.72 ± 35.53	176.16 ± 42.30	184.33 ± 39.96	180.10 ± 39.98 *
LDL (mg/dL)	Baseline	100.52 ± 35.18	109.20 ± 34.74	103.37 ± 34.31	103.92 ± 32.80	104.27 ± 34.20
	Week 10	101.13 ± 33.37	107.97 ± 30.86	103.84 ± 36.68	108.78 ± 36.85	105.41 ± 34.58
CRP (mg/L)	Baseline	1.92 ± 2.10	1.74 ± 1.74	1.21 ± 1.43	1.74 ± 1.78	1.63 ± 1.77
	Week 10	1.86 ± 2.13	2.16 ± 2.09	1.38 ± 1.70	1.71 ± 1.80	1.76 ± 1.94

Note: HFCS—High fructose corn syrup, LDL—Low Density Lipoprotein, CRP—C Reactive Protein. All Groups consumed sugar sweetened, low-fat milk in amounts that the added sugar contributed 9% (fructose and glucose groups) or 18% (sucrose and HFCS groups) of the calories required for the participant to maintain weight. * Significant difference pre *vs.* post in the entire study population *p* < 0.01; ** *p* < 0.001.
